# Staged endoscopic endonasal and bifrontal interhemispheric resection of a giant craniopharyngioma: a two-corridor maximal safe resection strategy

**DOI:** 10.3171/2026.7.FOCVID26121

**Published:** 2026-10-01

**Authors:** Daniele Starnoni, Alexander F. Kuffer, Danielle Golub, Mark B. Chaskes, Amir R. Dehdashti

**Affiliations:** 1 Department of Neurosurgery, North Shore University Hospital, Northwell Health, Donald and Barbara Zucker School of Medicine at Hofstra/Northwell, Manhasset; and; 2 Department of Otolaryngology Head and Neck Surgery, Northwell Health, New York, New York

**Keywords:** craniopharyngioma, endoscopic endonasal approach, bifrontal interhemispheric approach, transcallosal approach, maximal safe resection, skull base surgery

## Abstract

This video illustrates decision-making in modern maximal safe resection of a giant transinfundibular craniopharyngioma. The case highlights the selective use of complementary corridors to address distinct anatomical compartments while minimizing traction on critical neurovascular and hypothalamic interfaces. The first stage prioritizes urgent ventral decompression of the optic apparatus, third ventricle, and prepontine cistern; the second stage targets a fixed superior suprasellar/anterior third ventricular cystic component. The central lesson is that maximal safe resection is not defined by a single corridor, but by combining complementary routes to achieve durable resection while protecting critical hypothalamic, visual, and vascular interfaces.

The video can be found here: https://stream.cadmore.media/r10.3171/2026.7.FOCVID26121

## Transcript

This video demonstrates a staged endoscopic endonasal and bifrontal interhemispheric resection of a large craniopharyngioma in a 62-year-old woman who presented with progressive cognitive decline, behavioral changes, drowsiness, and gait disturbance.^1^

### 0:37 Preoperative Imaging.

Imaging by CT scan and MRI revealed what seemed to be a typical craniopharyngioma, very large in size, measuring more than 5 cm, partially cystic, with internal calcifications. As evident on this picture, there was obstructive hydrocephalus, periventricular transependymal CSF flow, and a true transinfundibular craniopharyngioma spanning multiple compartments.^2,3^

### 1:03 Rationale for Staged Surgery.

As a one-stage surgery could not safely address the whole tumor, we decided to perform a staged procedure. First, we performed an endoscopic endonasal transplanum approach to decrease the size of the tumor, remove the central portion, drain the cyst, and relieve mass effect on the optic chiasm and the medial regions of both frontal lobes. This was then followed by a craniotomy and bifrontal approach for complete resection.^4–7^

### 1:37 Endonasal Setup and Exposure.

Here is the setup for the endonasal approach, with harvest of the nasoseptal flap and placement of the flap in the nasopharynx or maxillary sinus, followed by exposure of the sphenoid sinus. We make sure that the flap is large enough, and then the transplanum exposure is performed here, with removal of the bone beyond the region of the superior intercavernous sinus and drilling of the sella.

The removal of the bone over the sella is really to give us access and maneuverability. The real exposure and resection will be done through the dura of the planum.^4–6^

### 2:32 Dural Opening and Cyst Decompression.

Here the dura is opened. As you can see, as soon as the dura is opened, the cyst is drained. Now the superior intercavernous sinus is being ligated and cut to connect the dura of the planum to the dura of the sella. Then we expose the dura all the way laterally to the medial opticocarotid recesses and start resecting this typical craniopharyngioma.^4–6^

### 2:46 Resection of Cyst Wall and Calcified Nodule.

As you can see, we remove the cyst wall and then the calcified part of the nodule, which is quite adherent to the optic chiasm. You can see the optic tract. You can see the entrance to the third ventricle. You can also see how the pituitary stalk is intimately involved with the tumor and is not preservable.^8,9^

### 3:09 Microsurgical Dissection Around the Basilar Apex.

Microdissection, in the same way that we do with microsurgical microscopic technique, is performed here. You can see the basilar artery, the branching of the PCA and SCA, and further removal of the tumor is performed by piecemeal resection, with preservation of the arachnoid, preservation of neurovascular structures, and, very importantly, preservation of the tiny perforators to the optic chiasm.

This is done very meticulously, with coagulation of direct feeders going to the tumor, while preserving the feeders to the optic chiasm. A nice plane is identified here. As evident, the tumor is very stuck to the posterior aspect of the optic chiasm and the anterior aspect of the basilar artery and basilar bifurcation.^8^

### 4:04 Critical Retrochiasmatic and Infrachiasmatic Dissection.

The tumor is now being removed from the most critical part, which is the top of the basilar artery. This is the area where inadvertent traction on the tumor can cause injury to the PCA or basilar artery and create bleeding or a pseudoaneurysm, which can sometimes become evident later on follow-up imaging.^8^

Here, we can see that there is a pristine plane that we are creating between the tumor and the basilar artery. There is a perforator from the basilar artery that is very stuck to the tumor and requires meticulous dissection and separation before ultimately removing the tumor from this retrochiasmatic, but mostly infrachiasmatic, portion.^8^

### 4:53 Lateral and Pericarotid Tumor Removal.

We then proceed with removal of the tumor on the more lateral aspect around the medial opticocarotid recess, which had been exposed during the bony exposure, and the tumor is further removed medial to the carotid artery. You can see the pulsation of the carotid artery on the left side, and the tumor from the medial and superior aspect of the carotid is being resected.^4–6^

You can see that extension of the dural opening laterally is possible because the medial opticocarotid recess has been fully drilled. I cannot overemphasize the importance of this bilaterally to give us lateral exposure and allow removal of the tumor in the pericarotid region.^4–6^

### 5:36 Endonasal Reconstruction and Result After Stage 1.

This completes the endonasal part, with good but subtotal resection of the tumor in the inferior and central portion, followed by fascia lata inlay and onlay grafts, nasoseptal flap, Surgicel sealant, glue, and NasoPore in the nostril.^4–6^

This already caused marked improvement in the patient’s confusion, drowsiness, and gait disturbance. Aside from the superior cystic component that you can see here, the rest of the tumor was removed, and the patient’s neurological examination almost returned to normal.

### 6:12 Second-Stage Bifrontal Interhemispheric Approach.

We brought the patient back a few weeks later, after dramatic improvement, for completeness of resection. This was performed through a coronal incision, bifrontal craniotomy, and interhemispheric approach to complete the resection of the superior part of the tumor cyst wall and whatever tumor remained.^1,7,10^

You can see here that a retractorless interhemispheric exposure is performed. The ACA branches are identified, and by further widening the space and going beyond the ACA and through the thin rim of the corpus callosum, we identify the cyst wall.^10^

### 6:52 Intraventricular Cyst Wall Dissection.

These are the pericallosal branches. After going through the corpus callosum, you can see the cyst wall. The cyst is opened and drained, and then we deal with the cyst wall, which is adherent to the ependyma of the ventricle.^8,10^

As this part of the tumor is basically inside the ventricle, microsurgical dissection is used to separate the cyst wall from the ependyma. Very minute dissection is performed to make sure that the ependyma remains intact, as well as the deeper branches of the anterior cerebral artery, because there are adhesions to those structures as well.^8,10^

### 7:28 Completion of Resection.

You can see that the more solid part of the cyst is being resected, while the rest is being peeled away. At the end, we are left only with the inflamed wall of the ventricular ependyma, without any residual cyst, after complete resection of the cyst wall.^8^

This is the final view, with closure of the dura in a watertight fashion, fat graft in the frontal sinus, and routine closure.

### 7:56 Postoperative Outcome.

Postoperative MRI shows complete resection of the tumor despite its complexity. The patient returned to her baseline function and remained neurologically intact, except for panhypopituitarism, which was appropriately treated with full hormone replacement. Follow-up MRI also showed stable gross-total resection, with no sign of residual disease.^1,9^

### 8:19 Key Surgical Lessons.

Key surgical lessons are summarized here. Again, we cannot overemphasize the importance of minute microsurgical dissection around the neurovascular structures in this very large craniopharyngioma, given the significant adhesions that exist.^1,8^

Thank you.

## Disclosures

The authors report no conflict of interest concerning the materials or methods used in this study or the findings specified in this publication.

## Author Contributions

Primary surgeon: Dehdashti. Assistant surgeon: Starnoni, Kuffer, Golub, Chaskes. Editing and drafting the video and abstract: Starnoni, Kuffer, Golub. Critically revising the work: Kuffer, Dehdashti. Reviewed submitted version of the work: Kuffer, Golub. Approved the final version of the work on behalf of all authors: Starnoni. Supervision: Dehdashti.

## Supplemental Information

### Patient Informed Consent

The necessary patient informed consent was obtained in this study.
